# Supplementary material for: The Impact of Statin Use on Sepsis Mortality: A Systematic Review and Meta-Analysis
Source: Medicina (Kaunas). 2025 Aug 30;61(9):1563. doi: 10.3390/medicina61091563 (PMC12471783; doi:10.3390/medicina61091563)
Supplement: Supplementary file 1 [file medicina-61-01563-s001.zip › medicina-3801447-supplementary.pdf]

Systematic Review

The Impact of Statin Use on Sepsis Mortality: A Systematic Review and Meta-Analysis

Constantinos Philippou [1], Constantinos Tsioutis [1,*], Maria Tsiappari [1], Nikolaos Spernovasilis [1,2], Dimitrios Papadopoulos [1] and Aris P. Agouridis [1,3]

1 School of Medicine, European University Cyprus, Nicosia 2404, Cyprus; cp211877@students.euc.ac.cy (C.P.); mt192099@students.euc.ac.cy (M.T.); nikspe@hotmail.com (N.S.); d.papadopoulos@euc.ac.cy (D.P.); a.angouridis@euc.ac.cy (A.P.A.)
2 Department of Infectious Diseases, German Medical Institute, Limassol 4108, Cyprus
3 Department of Internal Medicine, German Medical Institute, Limassol 4108, Cyprus
* Correspondence: k.tsioutis@euc.ac.cy

Supplementary material

Study or Subgroup	Statins users Events	Statins users Total	non-statin users Events	non-statin users Total	Weight	Risk Ratio M-H, Fixed, 95% CI	Year
Kruger 2006	7	66	86	372	3.9%	0.46 [0.22, 0.95]	2006
Thomsen 2006	35	176	1114	5177	10.9%	0.92 [0.68, 1.25]	2006
Martin 2007	6	16	18	37	1.6%	0.77 [0.38, 1.57]	2007
Dobesh 2009	19	60	62	128	5.9%	0.65 [0.43, 0.99]	2009
Mekontso Dessap 2011	9	44	13	32	2.2%	0.50 [0.25, 1.03]	2011
Fuller 2012	4	18	29	73	1.7%	0.56 [0.23, 1.39]	2012
Cuervo 2013	18	44	124	282	5.0%	0.93 [0.64, 1.36]	2013
Ajrouche 2013	42	106	149	245	13.4%	0.65 [0.50, 0.84]	2013
López-Cortés 2013	5	33	42	127	2.6%	0.46 [0.20, 1.07]	2013
Mansur 2015	12	26	38	88	2.6%	1.07 [0.66, 1.73]	2015
Zechmeister 2015	31	73	30	46	5.5%	0.65 [0.46, 0.92]	2015
Schmidt 2016	13	40	42	80	4.2%	0.62 [0.38, 1.01]	2016
Mullis 2016	108	344	236	592	25.8%	0.79 [0.65, 0.95]	2016
Caffrey 2017	19	141	33	141	4.9%	0.58 [0.34, 0.96]	2017
Wiewel 2018	48	194	67	194	10.0%	0.72 [0.52, 0.98]	2018
**Total (95% CI)**		**1381**		**7614**	**100.0%**	**0.73 [0.66, 0.80]**	
Total events	376		2083				

Heterogeneity: Chi² = 13.89, df = 14 (P = 0.46); I² = 0%
Test for overall effect: Z = 6.41 (P < 0.00001)

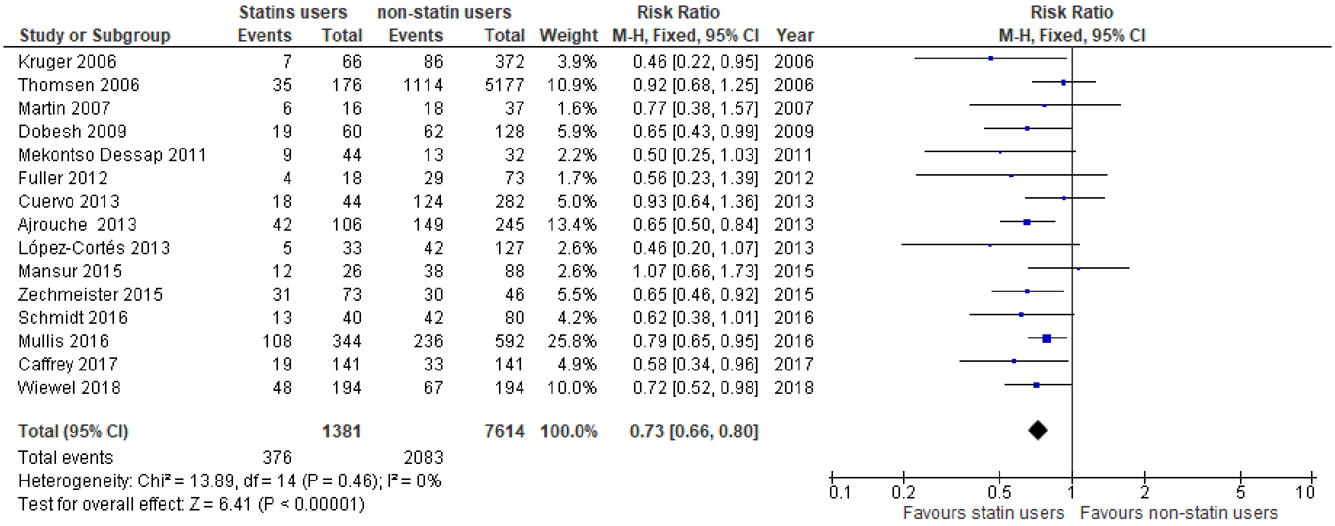

Supplementary Figure S1. Forest plot of comparison: Statin users vs non-Statin users in Cohort studies, after excluding the outlier studies.

	Statins users		non-statin users			Risk Ratio	
Study or Subgroup	Events	Total	Events	Total	Weight	M-H, Fixed, 95% CI	Year
Kruger 2006	7	66	86	372	0.4%	0.46 [0.22, 0.95]	2006
Thomsen 2006	35	176	1114	5177	1.0%	0.92 [0.68, 1.25]	2006
Martin 2007	6	16	18	37	0.2%	0.77 [0.38, 1.57]	2007
Yang 2007	20	104	66	350	0.4%	1.02 [0.65, 1.60]	2007
Dobesh 2009	19	60	62	128	0.6%	0.65 [0.43, 0.99]	2009
Goodin 2011	21	124	87	444	0.5%	0.86 [0.56, 1.33]	2011
Leung 2012	76	447	100	458	1.4%	0.78 [0.60, 1.02]	2012
Fuller 2012	4	18	29	73	0.2%	0.56 [0.23, 1.39]	2012
Cuervo 2013	18	44	124	282	0.5%	0.93 [0.64, 1.36]	2013
López-Cortés 2013	5	33	42	127	0.2%	0.46 [0.20, 1.07]	2013
Ou 2014	5253	27792	6059	27792	85.2%	0.87 [0.84, 0.90]	2014
Mansur 2015	12	26	38	88	0.2%	1.07 [0.66, 1.73]	2015
Schurr 2016	0	22	12	68	0.1%	0.12 [0.01, 1.95]	2016
Lee 2017	525	3325	581	3325	8.2%	0.90 [0.81, 1.01]	2017
Wiewel 2018	48	194	67	194	0.9%	0.72 [0.52, 0.98]	2018
**Total (95% CI)**		**32447**		**38915**	**100.0%**	**0.86 [0.84, 0.89]**	
Total events	6049		8485				

Heterogeneity: Chi² = 14.03, df = 14 (P = 0.45); I² = 0%
Test for overall effect: Z = 9.36 (P < 0.00001)

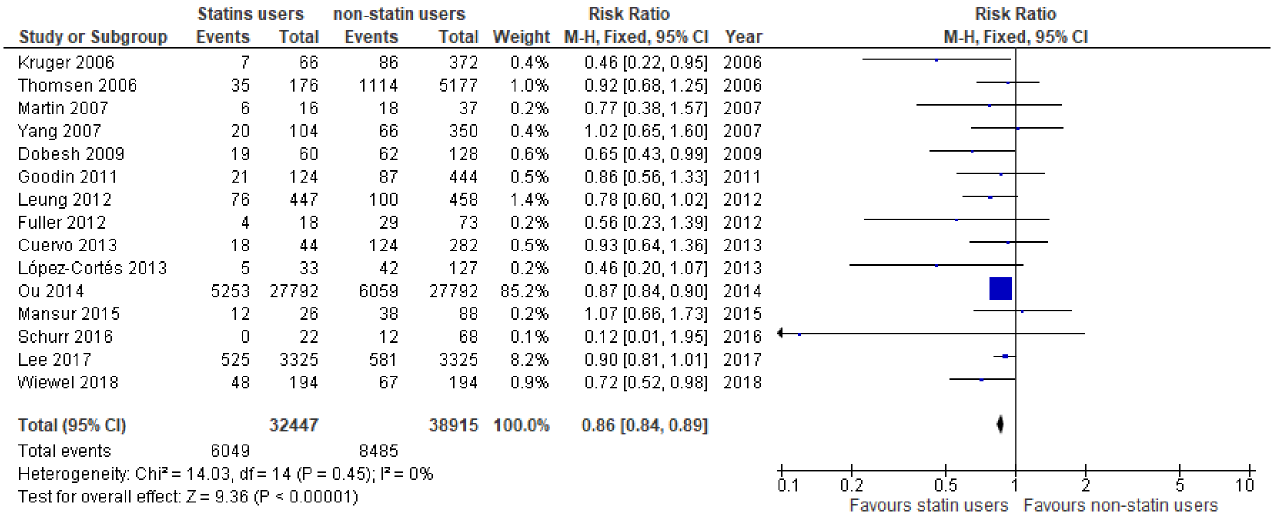

Supplementary Figure S2. Forest plot of comparison: prior statin use and continuation of statins vs non-statin use, after excluding the outlier studies.
